# Anti-inflammatory and nephroprotective activity of *Juglans mollis* against renal ischemia–reperfusion damage in a Wistar rat model

**DOI:** 10.1186/s12906-019-2604-7

**Published:** 2019-07-26

**Authors:** Jonathan Perez-Meseguer, Liliana Torres-González, Jorge Aurelio Gutiérrez-González, Gabriela Alarcón-Galván, Homero Zapata-Chavira, Noemi Waksman-de Torres, Diana Patricia Moreno-Peña, Linda Elsa Muñoz-Espinosa, Paula Cordero-Pérez

**Affiliations:** 1 Department of Analytical Chemistry, Universidad Autónoma de Nuevo León School of Medicine, Av Dr. Aguirre Pequeño and Madero S/N, Mitras Centro, C.P 64460 Monterrey, Nuevo León Mexico; 2 Department of Internal Medicine, Universidad Autónoma de Nuevo León, “Dr. José E. González” University Hospital Liver Unit, Av. Gonzalitos 235, Mitras Centro, C.P. 64460 Monterrey, Nuevo León Mexico; 3 Universidad Autónoma de Nuevo León, “Dr. José E. González” University Hospital Transplant Service, Av. Gonzalitos 235, Mitras Centro, C.P. 64460 Monterrey, Nuevo León Mexico; 4 Basic Science Department, Universidad de Monterrey, School of Medicine, UDEM, Av. Ignacio Morones Prieto 4500, C.P 66238 San Pedro Garza García, Nuevo León Mexico

**Keywords:** *Juglans mollis*, Oxidative stress, Ischemia–reperfusion, Antioxidant

## Abstract

**Background:**

Oxidative stress and the inflammatory process are involved in ischemia–reperfusion (I/R) injury. *Juglans mollis* has been reported as having antioxidant activity, which could attenuate the damage caused by I/R. We evaluated whether a methanolic extract of *Juglans mollis* (JM) exhibits nephroprotective activity in a Wistar rat model of I/R injury.

**Methods:**

Four groups of six rats were used: Sham, I/R, JM, and JM + I/R. Two groups were dosed with JM (300 mg/kg) for 7 days before I/R. I/R injury was induced by clamping the renal hilums for 45 min and then reperfusing the kidneys for 15 h. Blood samples were taken to evaluate the levels of alanine aminotransferase (ALT), blood urea nitrogen, creatinine, superoxide dismutase (SOD), malondialdehyde (MDA), interleukin 1β (IL-1β), IL-6, and tumor necrosis factor α (TNF-α).

**Results:**

The levels of creatinine, ALT, MDA, IL-1β, IL-6, and TNF-α were lower in JM + I/R than in I/R rats, whereas SOD level only was higher in JM + I/R than in Sham rats. No biochemical or histological damage was observed in JM rats compared with Sham rats; however, less histological damage was observed in JM + I/R rats compared with I/R rats.

**Conclusions:**

To our knowledge, this is the first report of nephroprotective activity of *J. mollis* against damage induced by I/R. This activity may be related to decreased levels of proinflammatory cytokines (IL-1β, IL-6, and TNF-α) and modulation of oxidative stress markers (SOD and MDA) observed in the present study.

## Background

Renal ischemia–reperfusion (I/R) injury is a major contributor to acute kidney failure. I/R injury results from temporary interruption of blood supply followed by recovery of blood flow, which leads to a profuse oxygenation state in the hypoxic renal tissue [1, 2]. I/R injury can occur for various reasons, including severe bleeding or when the renal blood flow is interrupted for some period, such as during renal transplantation or partial nephrectomy. Regardless of the cause, I/R injury can cause various degrees of damage to kidney tissue [3, 4]. The mechanisms of injury include the production of reactive oxygen species (ROS) and proinflammatory cytokines, and activation of multiple enzymes [3, 5, 6]. Diseases with multiple possible etiologies, such as I/R injury, involve the presence or generation of ROS, and the antioxidant system plays an important role in the course of the disease. For this reason, the use of antioxidant substances as potential agents for the treatment of diseases mediated by ROS has attracted worldwide interest [1, 2].

Various plants used in traditional medicine have been shown to have antioxidant activity in vitro and in vivo. Some drugs and natural compounds, and crude extracts of certain plants with antioxidant activity, have been evaluated to determine whether they are nephroprotective in rat I/R models [7, 8]. In this context, some species of the *Juglans* genus exhibit diverse biological activities that may be helpful in the treatment of diseases such as diarrhea, helminthiasis, sinusitis, arthritis, stomach pain, fever, eczema, diabetes mellitus, skin disorders, asthma, hepatitis, liver fibrosis, dysfunctional thyroid, anorexia, urinary tract infection, and cancer [9–13]. The *Juglans* genus has been reported to have antihypertensive activity [14–17] as well as antioxidant, lipolytic [18], antihyperglycemic, antilipidemic [19], neuronal cell-stimulating [20], and antiproliferative properties [21].

*Juglans mollis* belongs to the family *Juglandaceae*, commonly known as “*nogalillo*”, “*nuez encarcelada*”, or “*nogal blanco*”, and has been used in traditional medicine in northeastern Mexico for the treatment of infections, skin wounds, and ulcerations. Currently, the evidence for the biological activity of *J. mollis* is inconsistent. It has been reported that an extract of bark from *J. mollis* has potent antioxidant, hepatoprotective, and antimycobacterial activity [22–24]. In the present study, we evaluated the biological activity of *J. mollis* against renal damage induced by I/R in rats.

## Methods

### Plant material

Bark from *J. mollis* was collected in Villaldama, N.L., Mexico (26° 23′ 52.3068“ N, 100° 25’ 27.606” W), during summer of 2016*.* The plant was authenticated by Prof. Humberto Sánchez at the School of Biology of the Universidad Autónoma de Nuevo León and a voucher specimen was deposited in the institutional herbarium (voucher specimen no. UAN-2429). No harm was made to the tree during bark acquisition, since only a small amount was taken. The process did not require the use of roots. According to the institutional guidelines of the UANL, only the approval of a research ethics committee is required for the collection of the plant, since it is only for research purposes (HI17–00002). The bark of *J. mollis* was ground finely, and 300 g of the powdered bark was extracted by maceration and shaker agitation with methanol three times for 1 h each time. The supernatant was filtered and evaporated under reduced pressure at 37 °C, dried in an oxygen-free environment, and stored at 4 °C until use.

### Phytochemical analysis

The ethanolic extract of *J. mollis* underwent preliminary phytochemical screening to determine the presence of alkaloids (Dragendorff test), carbohydrates (anthrone test), carboxyl groups (sodium bicarbonate test), coumarins (Ehrlich test), flavonoids (Shinoda test), phenolic hydroxyls (ferric chloride test), quinones (Bornträger test), saponins (foam formation test), and sterols and triterpenes (Liebermann–Burchard test) [25].

### Animals

Experiments were performed using male Wistar rats weighing 200–300 g (purchased from Círculo A.D.N., México City). Animals were maintained at a stable room temperature (24 ± 3 °C) under a 12-h light–dark cycle and fed with commercial rat pellets (Prolab diet 2500, Nutrimix, México City) and water ad libitum. All animal procedures were performed in accordance with the proper use and care of laboratory animals, as approved by the ethics committee of our institution (HI17–00002) and according to the specifications of the Mexican Official Law NOM-062-ZOO-1999.

### Experimental design

To evaluate the activity of *J. mollis* extract in the rat model of renal I/R, the following experimental groups were used (*n* = 6 rats/group).

1. Control (Sham): rats were treated with saline solution for 7 days by oral administration (p.o.), after which they underwent a sham laparotomy without involving the renal pedicle.
2. *J. mollis* extract (JM): rats were treated with a 300 mg/kg dose of extract for 7 days, after which they underwent a surgical procedure identical to that undergone by the Sham rats.
3. I/R: rats were treated with saline solution for 7 days, after which acute kidney injury was induced by bilateral ischemia (45 min) followed by reperfusion (15 h).
4. *J. mollis* extract + I/R (JM + I/R): rats were treated with a 300 mg/kg/day dose of the extract for 7 days (p.o.) continuously before injury, after which, acute kidney injury was induced using the same procedure as that for the I/R group.

### Kidney injury

Rats were anesthetized using xylazine (Sedaject, Vedilab SA de CV, Querétaro, México Reg. SAGARPA Q-0088-122) intraperitoneally at a dose of 10 mg/kg of body weight with ketamine (Anesket, PiSA Agropecuaria SA de CV, Guadalajara, México, Ltd. Reg. SAGARPA Q7833–028) as an analgesic at a dose of 100 mg/kg according to the specifications of the Mexican Official Law NOM-062-ZOO-1999. The I/R and JM + I/R rats then received a laparotomy. Their kidneys were exposed and then subjected to 45 min of ischemia by occlusion of the renal pedicle, after which the clamps were removed and reperfusion was allowed for 15 h. During this period, the rats were allowed food and water ad libitum. Rats underwent anesthesia and blood samples were collected, all rats were euthanized by exsanguination and serum was stored at − 80 °C until use. For the Sham and JM rats, the procedure involved laparotomy as for the other groups but without any occlusion. This rat I/R model produces acute kidney damage, as shown by changes in oxidative and inflammatory stress markers, similar to that exhibited by the graft before a transplant in human patients [26].

### Biochemical analyses

Briefly, blood samples were centrifuged for 15 min at 3,500 rpm (SIGMA 2–5 Centrifuge, Osterode am Harz, Germany) and the serum was separated. Blood urea nitrogen (BUN), creatinine, and alanine aminotransferase (ALT) concentrations were measured using an ILab-300 Plus clinical chemical analyzer and commercial kits (both from Instrumentation Laboratory, Bedford, MA, USA). The levels of these substances were measured to exclude possible toxic effects of the *J. mollis* extract on the liver and kidney and to assess its effects on renal and hepatic function.

### Measurement of malondialdehyde (MDA) concentration

The extent of lipid peroxidation in renal tissue was estimated by measuring the MDA concentration colorimetrically using a TBARS assay kit (Cayman Chemical Company, Ann Arbor, MI, USA). The absorbance of adducts formed by the reaction of MDA and thiobarbituric acid was measured at 540 nm on a microplate-reading spectrophotometer (Thermo Scientific Multiskan FC, Waltham, USA) [27]. The results are expressed in terms of MDA equivalents in μmol/L.

### Measurement of superoxide dismutase (SOD) activity

SOD activity was measured using a commercial kit (Cayman Chemical Company) following the manufacturer’s instructions and is expressed in IU/mL. Briefly, 200 μL of radical detector, 10 μL of the tissue homogenate supernatant, and 20 μL of xanthine oxidase were added to the reaction mixture and, after 20 min, the absorbance of the mixture was measured at 460 nm using a microplate-reading spectrophotometer (Thermo Scientific Multiskan FC).

### Measurement of serum cytokine concentrations

The serum concentrations of the inflammatory cytokines interleukin 1β (IL-1β), IL-6, and tumor necrosis factor α (TNF-α) were measured using a sandwich enzyme-linked immunoassay development kit specific to that cytokine (PeproTech, Mexico City, Mexico). Avidin and avidin–peroxidase were added to produce a colorimetric end product, whose optical density was proportional to the concentration of the cytokine of interest. The optical density was measured at 405 and 620 nm using a microplate-reading spectrophotometer (Thermo Scientific Multiskan FC). The results are expressed as ng/mL.

### Histological analysis

Kidneys were resected and fixed in a 10% formaldehyde solution, embedded in paraffin, and cut into 5-μm sections, which were deparaffinized, hydrated, and stained with hematoxylin and eosin. The sections were then evaluated for indicators of cell damage as previously described with some modifications [28–30]. The histopathological changes analyzed were tubular necrosis, proteinaceous casts, exfoliated cells in the lumen, Bowman’s space enlargement, lymphocytes in peritubular capillaries, medullary congestion, and intracellular vacuolization.

Tubular necrosis, proteinaceous casts, exfoliated cells in the lumen, Bowman’s space enlargement, and lymphocytes in peritubular capillaries were graded as follows: absent (0); mild (1, unicellular, patchy isolated changes or changes in 1–4% of the observed sample); moderate (2, changes in 5–24% of the sample); severe (3, changes in 25–50% of the sample); and very severe (4, changes in > 50% of the sample).

The degree of medullary congestion was defined as follows: no congestion (0); mild (1, vascular congestion and identification of erythrocytes at × 40 magnification); moderate (2, vascular congestion and identification of erythrocytes at × 20 magnification); severe (3, vascular congestion and identification of erythrocytes at × 10 magnification); and very severe (4, vascular congestion and identification of erythrocytes at × 4 magnification).

The degree of intracellular vacuolization or tubular epithelial edema was defined as follows: absent (0), focal cellular edema (1), mild diffuse cellular edema (2), moderate diffuse cellular edema (3), and severe diffuse cellular edema (4).

### Statistical analysis

All data are expressed as the mean ± standard deviation of the mean. The data were analyzed using one-way analysis of variance with the Tukey post hoc test for parametric data or the Kruskal–Wallis test with a Dunn post hoc test for nonparametric data. Prism software (version 6.0; GraphPad, San Diego, CA, USA) was used for the analyses. Differences between means were considered significant at *p* < 0.05.

## Results

### Phytochemical profile

The phytochemical profile of *J. mollis* is shown in Table 1. All the functional tests were positive except for carboxyl groups and saponins.

**Table 1:** Phytochemical analysis of *Juglans mollis*

Chemical type	*J. mollis*
Alkaloids	+
Carbohidrates	+
Carboxyl group	–
Coumarines	+
Flavonoids	+
Phenolics	+
Saponins	–
Sterols and triterpens	+
Quinones	+

+, positive; −, negative

### Biochemical markers

**Fig. 1:**
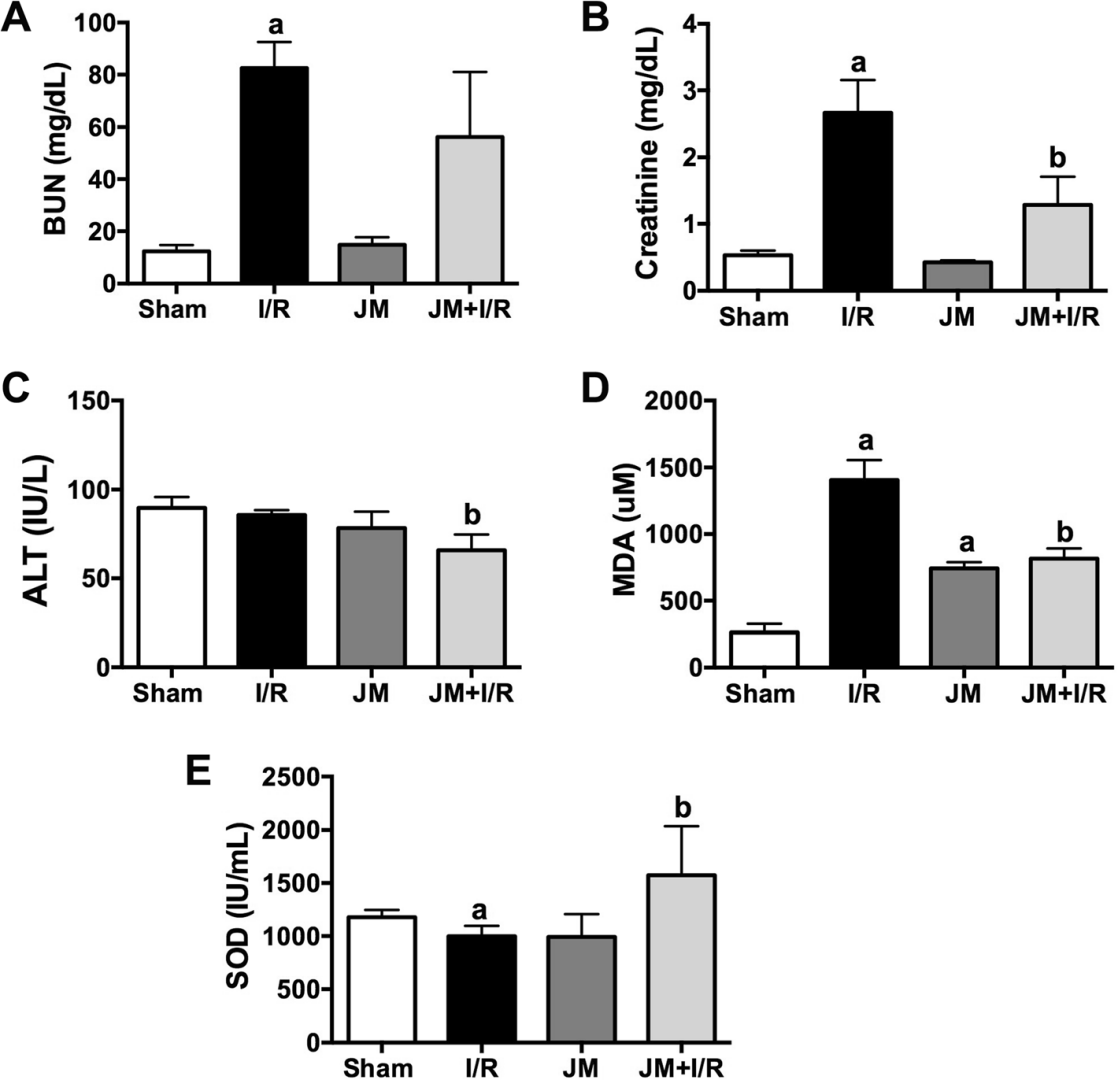
Changes in (**a**) BUN (**b**) Creatinine, (**c**) ALT, (**d**) MDA, and (**e**) SOD levels after I/R injury. Values are mean ± SD. a. *p* < 0.05 comparison with the sham group; b. *p* < 0.05, comparison with IR group

Serum BUN concentration was higher in I/R rats than in Sham rats (83.00 ± 10.00 mg/dL vs. 12.00 ± 2.00 mg/dL, respectively) but did not differ between JM (15.00 ± 3.00 mg/dL) and Sham rats. Serum BUN concentration was lower in JM + I/R rats than in I/R rats, but the difference was not significant (62.00 ± 26.00 mg/dL vs. 83.00 ± 10.00 mg/dL) (Fig. 1 a). Creatinine concentration was normal and did not differ between Sham and JM rats (0.53 ± 0.07 mg/dL vs. 0.42 ± 0.03 mg/dL, respectively). Creatinine concentration was significantly higher in I/R rats (2.66 ± 0.48 mg/dL) than in Sham, JM, and JM + I/R rats (1.28 ± 0.42 mg/dL) (Fig. 1 b). Serum ALT concentration, an indicator of liver damage, did not differ between Sham, I/R, and JM rats (89.00 ± 6.00 IU/L, 86.00 ± 3.00 IU/L, and 78.00 ± 9.00 IU/L, respectively); however, serum ALT concentration was significantly lower in JM + IR rats (66.00 ± 9.00 IU/L) than in I/R or Sham rats (Fig. 1 c).

### Oxidative stress markers

Tissue MDA concentrations were significantly higher in I/R rats (1405.00 ± 151.00 mM) than in Sham rats (263.00 ± 64.00 mM), JM rats (742.00 ± 46.00 mM, and JM + I/R rats (816.00 ± 75.00 mM). However, MDA concentration did not differ significantly between JM + I/R and JM rats (Fig. 1 d).

SOD concentration was significantly lower in I/R rats than in Sham rats (998.00 ± 98.00 IU/mL vs. 1178.00 ± 67.00 IU/mL, respectively), but not compared with JM rats (992.00 ± 216.00 IU/mL). Interestingly, SOD concentration was significantly higher in JM + I/R rats (1573.00 ± 463.00 IU/mL) than in Sham rats (Fig. 1e).

### Serum cytokines

**Fig. 2:**
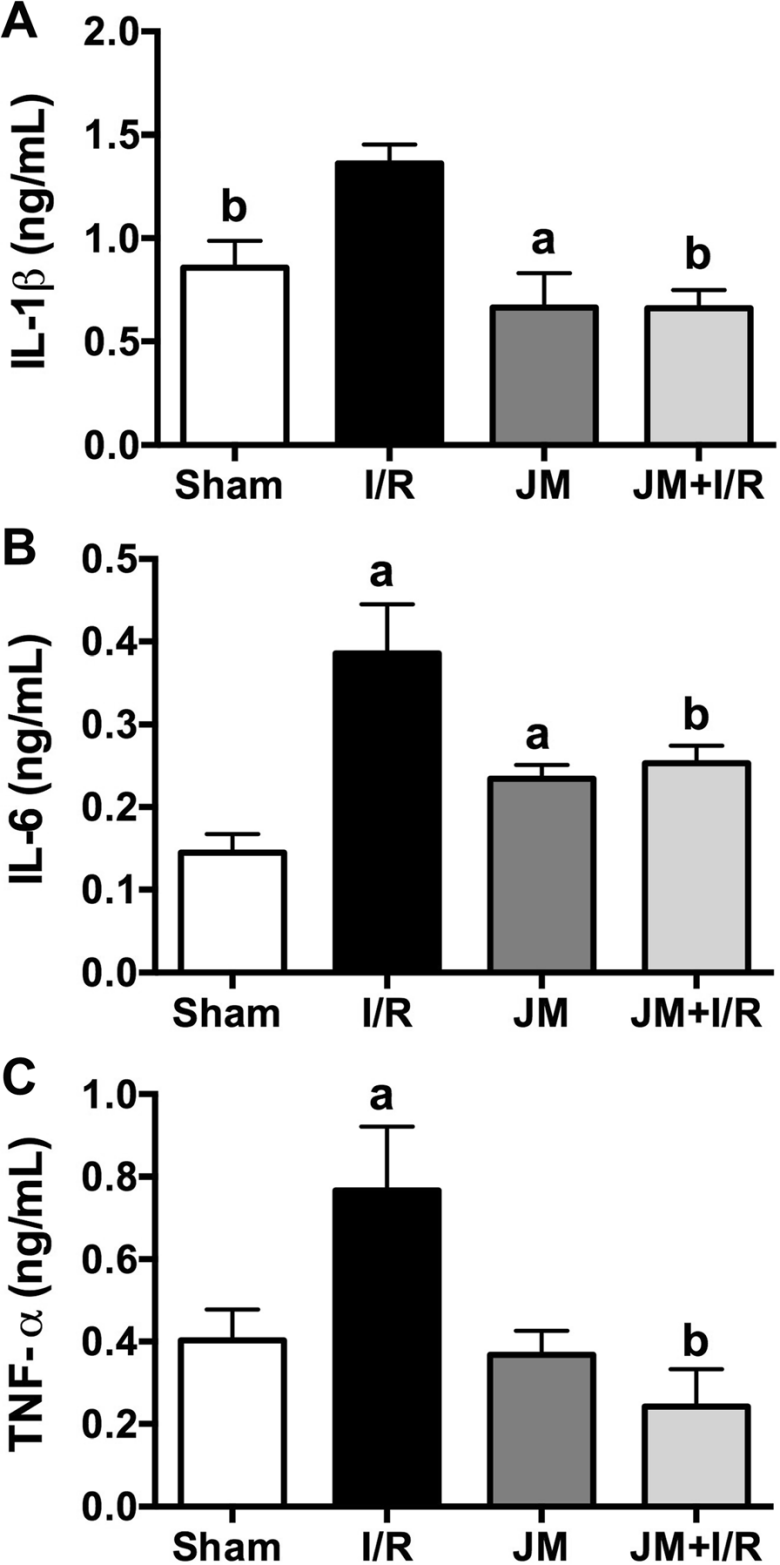
Serum concentrations of the proinflammatory cytokines in the various experimental groups. Data are presented as mean ± SD. **a**. *p* < 0.01, comparison with the sham group; **b**. *p* < 0.001, comparison with IR group

The concentrations of the three cytokines tested (IL-1β, IL-6, and TNF-α) were higher in I/R rats (1.36 ± 0.08 ng/mL, 0.39 ± 0.05 ng/mL, and 0.70 ± 0.04 ng/mL, respectively) than in Sham rats (0.86 ± 0.12 ng/mL, 0.14 ± 0.02 ng/mL, and 0.40 ± 0.07 ng/mL, respectively). Only IL-6 concentration was higher in JM rats (0.23 ± 0.01 ng/mL) than in Sham rats. The concentrations of the three proinflammatory cytokines were significantly lower in JM + I/R rats (0.66 ± 0.08 ng/mL, 0.25 ± 0.02 ng/mL, and 0.26 ± 0.07 ng/mL, respectively) than in I/R rats (Fig. 2).

### Histology

**Fig. 3:**
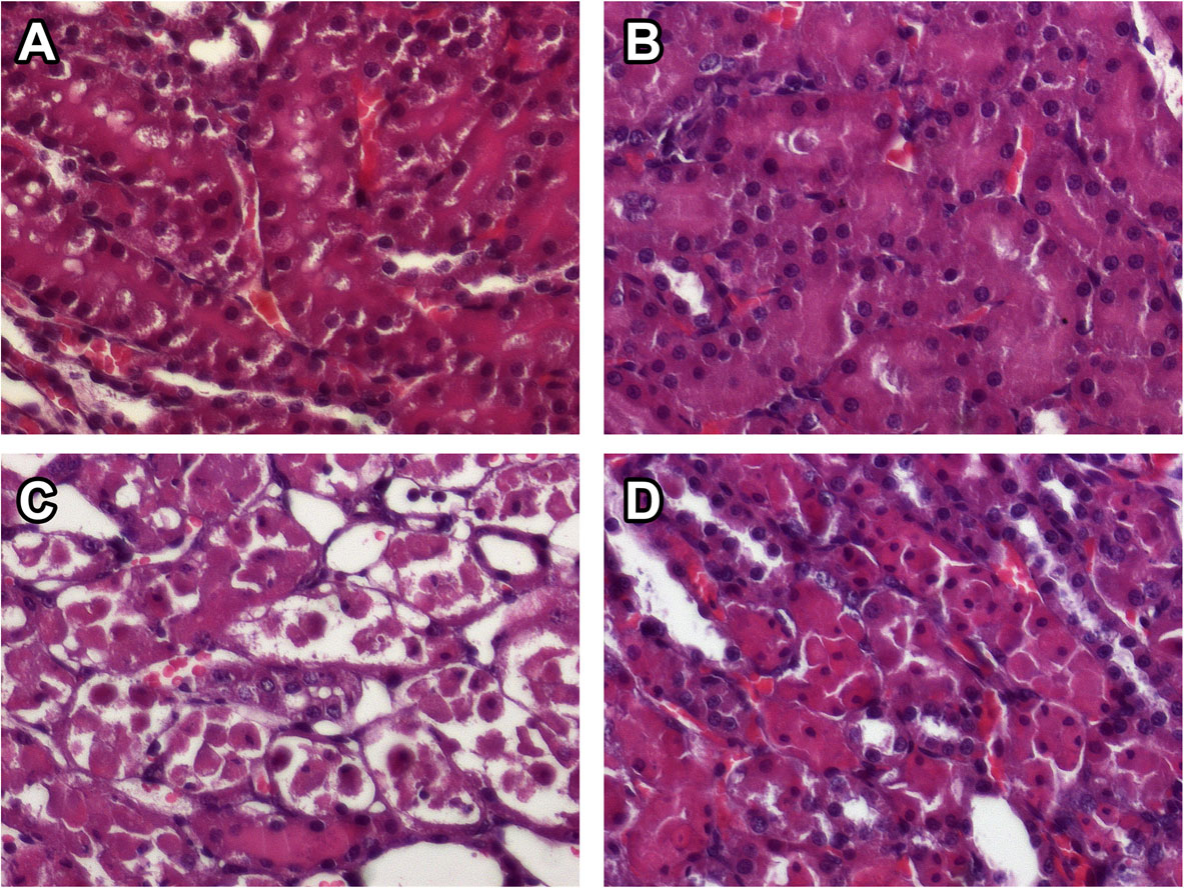
Histological sections of renal tissue × 40 (H&E). Sham (**a**), JM (**b**), I/R (**c**), and JM + I/R (**d**)

Kidneys from the Sham rats showed tubules, glomeruli, and a generally preserved structure. I/R kidneys exhibited dilatation and tubular necrosis in a generalized diffuse band in the medulla. In I/R kidneys, the cortex was preserved only in the lower part, protein cylinders were seen in the tubular lumen, and generalized diffuse necrosis was apparent. The JM kidneys also showed a preserved cortex, medulla, and general structure, and had an imperceptible inflammatory infiltrate. In the JM + I/R kidneys, injury was seen only in certain sections of the medulla, tubular dilatation and necrosis appeared focally, and ghost cells were observed between apparently preserved cells (Fig. 3). The histological parameters evaluated are shown in Table 2. The extent of tubular necrosis, medullary congestion, proteinaceous casts, and Bowman’s space enlargement differed significantly between IR and JM + IR rats.

**Table 2:** Histopathological changes in all studied groups

Experimental group	Intracellular vacualization	Medullary congestion	Proteinaceous casts	Exfoliated cells in the lumen	Tubular necrosis	Lymphocytes in peritubular capillares	Bowman’s space enlargement
Sham	1.17 ± 0.41	0.17 ± 0.41	0.67 ± 0.82	0.17 ± 0.41	0.00 ± 0.00	0.67 ± 0.52	0.33 ± 0.52
I/R	2.20 ± 0.45^a^	3.83 ± 0.41^a^	3.60 ± 0.55^a^	4.00 ± 0.00^a^	4.00 ± 0.00^a^	1.00 ± 0.00	2.80 ± 0.45^a^
JM	1.00 ± 0.00	1.00 ± 0.71	0.20 ± 0.45	0.40 ± 0.55	0.00 ± 0.00	1.00 ± 0.00	0.40 ± 0.55
JM + I/R	2.00 ± 0.00	0.50 ± .83^b^	2.00 ± 0.71^b^	2.80 ± 0.45^b^	2.00 ± 0.00^b^	1.00 ± 0.00	0.60 ± 0.89^b^

Values represent the mean ± S.D. (n = 6) of histopathological grade. Grade: no change (0), mild (1), moderate (2), severe (3), very severe (4). a. *p* < 0.05, comparison with the sham group; b. *p* < 0.05, comparison with IR group

## Discussion

During the I/R process, ROS accumulate, and this accumulation of ROS can lead to cell dysfunction and even cell death [31]. Various enzymes with antioxidant activity, such as SOD, participate in the neutralization of ROS [32]. However, under oxidative stress, the imbalance between the production of ROS and the ability of enzymes to counteract these oxidative radicals can lead to significant tissue damage and may be irreversible [24]. Many plants are recognized as a source of nonenzymatic antioxidants capable of attenuating oxidative damage induced by ROS [33].

Several species of the *Juglans* genus, such as *J. regia*, *J. mandshurica*, and *J. microcarpa*, have been shown to have substantial antioxidant activity in both in vivo and in vitro models [9, 13, 18]. Some polyphenols isolated from these species have been shown to have hepatoprotective and nephroprotective effects by increasing the activities of SOD and catalase [34, 35], which may mediate the plants’ antioxidant activities.

In this study, we assessed whether a methanolic extract of *J. mollis* has nephroprotective effects. We found that the oxidative stress damage induced by I/R process in JM + I/R rats was attenuated when the SOD concentration was increased. Previous studies by our group have identified an important antioxidant activity of *J. mollis* [22, 24] that may be involved in protecting against I/R damage. By contrast, the concentration of MDA, another marker of oxidative stress whose tissue levels increase in association with tissue damage, was lower in JM + I/R rats. This observation is consistent with the findings of a study about the effects of another species of *Juglans* (*J. mandshurica*) on the inhibition of peroxidation of lipids, which reported increased SOD and glutathione peroxidase levels, and decreased MDA level [36]. By contrast, MDA concentration was slightly higher in JM rats than in Sham rats. Various compounds such as anthocyanins (present in other species of *Juglans*) are interfering compounds that absorb at 532–540 nm. This may contribute to an overestimation of the MDA concentration, which may explain the higher concentration of this mediator of oxidative stress in the JM group [37–39].

In the present study, *J. mollis* extract exhibited functional groups that are characteristic of antioxidant compounds such as coumarins, flavonoids, phenolics, quinones, and carbohydrates (commonly linked to flavonoids). Terpenes and alkaloids were also present. These polyphenolic compounds may be responsible for the nephroprotective and antioxidant activities found here and the hepatoprotective effects reported previously [22, 24].

No toxicity has been reported for extracts obtained from the genus *Juglans* at the doses evaluated in a biological study [40]. We also found that *J. mollis* extract at the doses tested did not increase the concentrations of markers of hepatotoxicity (ALT and AST) and nephrotoxicity (BUN and creatinine), as shown by the lack of difference between these levels in JM and Sham rats.

Th1-type CD4^+^ T lymphocytes secrete proinflammatory cytokines such as TNF-α, IFN-γ, IL-2, and IL-12, whereas Th2-type CD4^+^ T lymphocytes secrete anti-inflammatory cytokines such as IL-4, IL-5, IL-10, and IL-13. Th2 cells exhibit seem to protect against I/R damage, whereas Th1 cells have deleterious effects [41–43], which suggests that an imbalance between the activities of these cell types can influence the extent of I/R lesions in the kidney during the transplantation process. For example, a large increase in the production of proinflammatory markers such as IL-1β, IL-6, and monocyte chemotactic protein 1 has been observed during recovery immediately after kidney transplantation [44].

Immunostimulating agents can be isolated from plants of the *Juglans* genus (*J. major* [45] and *J. jamaicensis* [46]). Compounds with immunostimulatory activity, such as the polysaccharide JRP1, which has an immunostimulatory effect in vivo by increasing the release of IFN-γ and IL-2, have been isolated from other species, including *J. mandshurica* [47, 48]. *J. regia* has been reported to have anti-inflammatory [49], antibacterial [50, 51], and antidiabetic [52, 53] properties. An extract of *J. regia* can inhibit nuclear factor κB (NF-κB) induced by TNF-α [54] and is an astringent because of its tannin content [55]. In the present study, I/R rats had high serum concentrations of the three proinflammatory cytokines IL-1β, IL-6, and TNF-α, whereas the JM + I/R rats had significantly lower concentrations of these three cytokines compared with the I/R rats. The concentrations of IL-1β and TNF-α were similar in JM and Sham rats, but IL-6 concentration was significantly higher in JM than in Sham rats. Similar effects have been reported for mononuclear cells from healthy people treated with a diet of olive oil and nuts, who showed a reduction in the postprandial response of proinflammatory cytokines with the exception of IL-6 [56].

Generalized damage along with tubular necrosis was observed as the formation of proteinaceous casts in the cortex and medulla in rat kidneys subjected to I/R injury. More structures were preserved and more focal tubular necrosis was observed at certain points in the medulla in JM + I/R rat kidneys. Kidneys from Sham and JM rats did not show any damage. In this study, several structural changes were observed in the renal tissue of JM + I/R rats, including decreasing tubular necrosis, medullary congestion, proteinaceous casts, and Bowman’s space enlargement, which is consistent with our results of biochemical, inflammatory and oxidative stress markers. These findings suggest that, at the histological level, *J. mollis* extract had a nephroprotective effect in rats subjected to I/R injury.

## Conclusions

To our knowledge, this is the first report of the nephroprotective activity of *J. mollis* against renal damage induced by I/R. This effect may be related to decreased production of proinflammatory cytokines (IL-1β, IL-6, and TNF-α) and modulation of oxidative stress markers (SOD and MDA) observed in the present study. The substantial antioxidant activity reported previously for this plant species may also be involved. Bioguided assays are needed to identify the compounds responsible for the activity of the methanolic extract of *J. mollis*.

## Data Availability

The datasets supporting the conclusions of this article are included within the article.

## Abbreviations

°C: celsius degrees
ALT: Alanine aminotransferase
BUN: Blood urea nitrogen
dL: deciliter
g: grams
h: hours
h: hours
H&E: hematoxylin and eosin
I/R: Ischemia/reperfusion
IL: Interleukin
IU: International unit
JM: *Juglans mollis* extract
kg: kilograms
MDA: malondialdehyde
mg: milligrams
min: minutes
mM: millimoles
ng: nanograms
nm: nanometers
p.o.: oral administration
ROS: Reactive oxygen species
SOD: Superoxide dismutase
TNF: Tumor necrosis factor
U/L: Units per liter
uL: microliters
um: micrometer
